# P20 Molecular characterization of *Staphylococcus aureus* from healthy humans and animals identifies within-host diversification and varied antibiotic pressure

**DOI:** 10.1093/jacamr/dlag102.026

**Published:** 2026-06-26

**Authors:** Idris Nasir Abdullahi, Carmen Lozano, Myriam Zarazaga, Carmen Torres

**Affiliations:** Department of Medical Laboratory Science, Faculty of Allied Health Sciences, College of Medical Sciences, Ahmadu Bello University, Zaria, Nigeria; Conjugation in Gram-positive Bacteria, Microbes in Health and Welfare, Centro de Biología Molecular Severo Ochoa (CSIC-UAM), C. Nicolás Cabrera 1, Universidad Autónoma de Madrid, Cantoblanco, 28049 Madrid, Spain; Area of Biochemistry and Molecular Biology, OneHealth-UR Research Group, University of La Rioja, Logroño, Spain; Area of Biochemistry and Molecular Biology, OneHealth-UR Research Group, University of La Rioja, Logroño, Spain; Area of Biochemistry and Molecular Biology, OneHealth-UR Research Group, University of La Rioja, Logroño, Spain; Area of Biochemistry and Molecular Biology, OneHealth-UR Research Group, University of La Rioja, Logroño, Spain

## Abstract

**Background:**

Understanding the within-host lineage and antimicrobial resistance (AMR) diversities of *Staphylococcus aureus* could provide insight into the dynamics of AMR crisis and effective infection preventive and control.

**Objectives:**

To determine the frequencies and diversity, perform molecular typing and compare the pattern of MDR in *S. aureus* from the nasal cavities of healthy humans and animals in Spain.

**Methods:**

Nasal samples were collected from the 52 nestling storks (NS), 57 humans who had no contact with animals (HH), 40 pigs (HP), and 10 pig farmers (HPF) from 4 pig farms, as well as 34 dogs (HD) and 41 dog owners (HDO) from 27 dog-owning households. The samples were processed to isolate and identify *S. aureus* by MALDI-TOF-MS. The antimicrobial susceptibility profile of all *S. aureus* strains was determined, and the genetic lineages of non-duplicated strains (from different samples or those of the same sample but with different AMR phenotypes) were studied by PCR/sequencing.

**Results:**

Nasal *S. aureus* carriage was identified in 36.5% NS, 36.8% HH, 65% HP, 80% HPF, 34.1% HDO, and 5.9% HD. Genetically diverse *S. aureus* strains (within-host multiple lineages and/ or AMR genotypes) were identified in 15.8%, 19%, 34.6%, 75%, and 64% of NS, HH, HP, HPF, and HDO carriers, respectively. Based on the number of clonal complexes (CCs) of *S. aureus* strains, significantly diverse CCs were obtained in NS of parents foraging in landfills (13 CCs) and the least in HP and HPF (2CCs) (*P<*0.005). Concerning methicillin-resistance trait and genetic lineages, most of the *S. aureus* from pigs (60%, CC398), pig farmers (70%, CC398), and one from a dog owner (7.1%, CC5) were methicillin-resistant *S. aureus* (MRSA. Whereas all others from the four hosts were methicillin-susceptible *S. aureus* (MSSA), of which the MSSA-CC398 was the predominant lineage. *S. aureus* strains from the HP and HPF presented the highest levels of MDR phenotype compared to other hosts, of which the least MDR strains were from NS (*P<*0.05).

**Conclusions:**

Relatively similar nasal *S. aureus* carriage was identified in all healthy humans (HH and HDO), except pig farmers, which reflects the impact of pigs’ nasal bacteria contaminating the farmers. The differences in the MDR rates present in *S. aureus* strains across the hosts revealed the variations in antibiotic pressure and the influence of animal contact. The high level of within-host diversification of *S. aureus* strains highlights the need for enhanced multiple sampling to obtain accurate data for the control of *S. aureus* and AMR transmission along the ‘One Health’ niches.

